# An innervated skin 3D in vitro model for dermatological research

**DOI:** 10.1007/s44164-022-00021-0

**Published:** 2022-06-10

**Authors:** Emma Rousi, Afonso Malheiro, Abhishek Harichandan, Ronny Mohren, Ana Filipa Lourenço, Carlos Mota, Berta Cillero-Pastor, Paul Wieringa, Lorenzo Moroni

**Affiliations:** 1 Department of Complex Tissue Regeneration, MERLN Institute for Technology Inspired Regenerative Medicine, Maastricht, The Netherlands; 2 Institute of Clinical Medicine, Department of Surgery, University of Turku, Turku, Finland; 3 M4i Division of Imaging Mass Spectrometry, Maastricht University, Maastricht, The Netherlands

**Keywords:** 3D in vitro models, Skin, Innervation, Screening, Biomimicry

## Abstract

A 3D in vitro model of innervated skin would be a useful tool in dermatological research to study the effect of different chemicals and compounds on the sensory properties of skin. Current innervated skin models are limited in composition and often composed of ex vivo skin explants and/or animal-derived material. In this study, our aim was to develop a human innervated skin model with a better biomimicry composition for in vitro research. Fibrin hydrogel and aligned electrospun fibers of poly(ethylene oxide terephthalate)/poly(butylene terephthalate) (PEOT/PBT) were used as a scaffold to generate the 3D in vitro model. The skin component was made of primary human keratinocytes and primary human fibroblasts, while the neuronal component was composed of iPSC-derived sensory neurons. Our results showed that the dermal component consisted of fibroblasts and synthesized collagen. The epidermal component was characterized by the expression of keratins 10 and 14, and involucrin. Finally, sensory neurons extended axons throughout the scaffold and reached the epidermis. Treating the model with a capsaicin solution for 30 min, which was performed as a proof of concept test for sensitization studies, resulted into partial depletion of substance P and tubulin β3. This model could be used for studying skin-neuron interactions and cutaneous toxicity.

**Supplementary Information:**

The online version contains supplementary material available at 10.1007/s44164-022-00021-0.

## Introduction

The need for applicable innervated skin tissue 3D in vitro models is acknowledged in the field of biomedical sciences already for several years to conduct tests in human tissue in a safer manner, study skin biology and physiology in health and disease conditions, and move away from animal testing [1]. Despite the rapid development in skin tissue engineering, skin is a complex organ, and in vitro models recapitulating its entire complexity have been proven challenging to engineers [2, 3]. When bioengineered skin substitutes are used in the clinics for treating burns, they are surrounded by their natural environment, and the healing and the maturation of the tissue, including the keratinization process for example, are completed by the body itself [4]. In the laboratory, this setting is challenging to replicate. Therefore, synthetic skin models often present with incomplete epidermis stratification or keratinization despite otherwise promising results [2, 3, 5].

An in vitro model combining skin and neurons would be useful in various research fields, such as in dermatology, neurosciences, and toxicity testing. In particular, the inclusion of sensory neurons in a skin model would allow for studying, e.g., skin sensitization when applying new cream formulations, or unraveling fundamental mechanisms of diseases. Also, neuronal-triggered reactions, such as pain, itching, allergy, skin inflammation, and regeneration, are relevant in dermatology practice and research [6–9]. However, there are currently not many available innervated skin models reported in the literature, and these are often composed of ex vivo skin explants and/or animal cells in the model, and during the cell culture phase [1, 10–12]. When developing an in vitro model for drug testing for humans, using only human cells would be a great advantage because of their better predictive capacity. Also, ex vivo skin can be challenging to obtain if a high number of samples are needed.

The requirements for in vitro skin models have been approached from different perspectives. Lee et al. [13] recently reported a complex system of hair-bearing human skin. However, these models based on human embryonic and induced pluripotent stem cells (iPSCs) required long culture period. Adding complexity into a skin model in the form of immune and neuronal components has also been performed [14, 15]. Development of a skin model for studying itch sensation in the context of atopic dermatitis has been described by Guo et al. [16]. The keratinization process of the epidermis has proven challenging to achieve in vitro, also in more complex skin models [14, 16]. Interestingly, the neuropeptides released by porcine neurons within an innervated skin model seemed to increase epidermal thickening [12].

Different biomaterials have been used as scaffolds for in vitro skin models. Collagen-based hydrogel solutions have successfully supported skin cells, but their shrinkage over time is inconvenient with regard of barrier function testing, among others [12, 15]. Combining collagen with silk has shown to lessen the shrinkage of the scaffold [14]. Recombinant spider silk protein has also been investigated as a scaffold for 3D in vitro models, but it still requires a significant amount of processing [17–19]. Cross-linked porous gelatin microbeads have also been used, yet without solving shrinkage challenges [3, 20]. A neuronal in vitro platform described by our research group comprised aligned electrospun PEOT/PBT fibers, fibrin hydrogel, and iPSC-derived sensory neurons, which showed to support neuronal growth. Thus, adding a skin compartment into this neuronal platform would be an interesting approach to fabricate a human 3D in vitro model of innervated skin [21].

Sensory neuron phenotype varies according to the innervating organ. These variances can be demonstrated, for example, by changes in the transient receptor potential (TRP) channel composition [22]. Along with histological and immunohistochemical affinity to native skin, the neurons’ ability to carry out a sensory stimulus is a key element for successful realization of an in vitro innervated skin model. This function could be studied by stimulating nociceptive neurons in the platform topically with capsaicin, a TRPV1 agonist, since the neurons in the native skin and the developed iPSC-derived neurons are known to express TRPV1 channel [21, 22]. Capsaicin cream with a high (8%) concentration is already used in the clinics to treat peripheral neuropathic pain by administering it percutaneously for 30 or 60 min [23, 24]. This treatment results in the depletion of the neuroinflammation-associated neuropeptide substance P from the neurons of the apical parts of the skin. However, this effect has also been noted to result from the loss of the nerve terminals from the epidermis, and not only from the changes in the neuropeptide release [24, 25].

Our aim was to develop a fully human innervated skin 3D in vitro model with dermal and epidermal components, and a neuronal component of iPSC-derived neurons cultured on electrospun PEOT/PBT fiber-based platform [21]. The iPSC neurons were seeded as single spheroids to mimic the anatomy of a sensory neuron with axons sprouting from it towards the innervated organ. Mass spectrometry imaging (MSI) was applied to show the suitability of this skin model for MSI analysis, e.g., the absorption of compounds, as previously shown for commercially available skin substitutes [26]. The innervated skin model reported here could facilitate in vitro drug development and dermatological research, and it would be also applicable for the cosmetic industry.

## Materials and methods

### Electrospinning

**Fig. 1:**
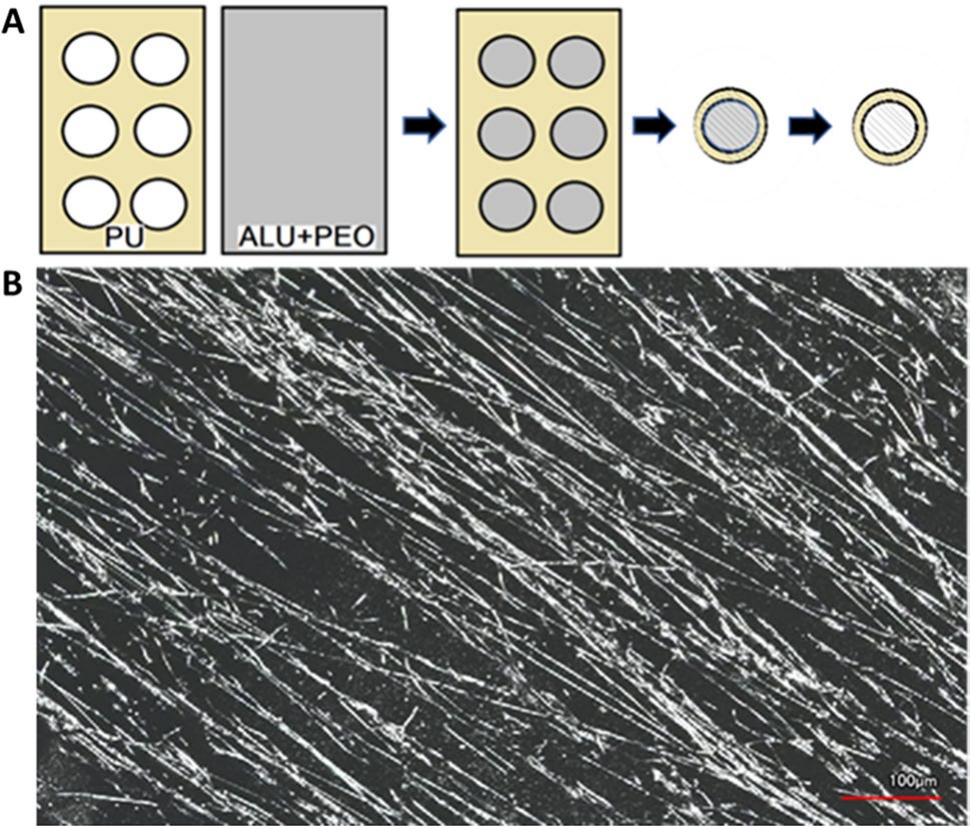
**A** Schematic presentation of the electrospinning process. **B** Profilometer picture shows the alignment of the electrospun nanofibers. Scale bar: 100 μm

Electrospun scaffolds were produced by punching a polyurethane (PU) mesh with a 6-mm round punch. After this, a piece of aluminum foil with the same size was fastened around a rotating mandrel, and the aluminum foil was electrosprayed with polyethylene oxide (PEO, 189,456, Sigma-Aldrich, MO, USA). After electrospraying, the punched PU mesh was inserted on top of the aluminum foil to the mandrel and polyactive (PA; 300PEOT55PBT45, PolyVation, Groningen, The Netherlands) was electrospun on top of the mesh to create aligned nanofibers. Finally, the scaffolds were punched with an 8-mm punch concentric to the 6-mm holes in the PU. Before placing the scaffolds into the cell culture insert, the PEO was leached out with phosphate-buffered saline (PBS) solution. The scaffolds were disinfected in a 48-well plate by soaking with 70% ethanol for 30 min and allowed to air dry for 45 min in a sterile flow cabinet. The scaffolds were imaged with a VK-X Optical Profilometer and the images were processed using VK-X MultiFile Analyzer (Keyence, Osaka, Japan). The scaffold production process is visualized in Fig. 1.

### Cell culture

Neonatal normal human epidermal keratinocytes (NHEKneo) and neonatal human dermal fibroblasts (NHDFneo) were purchased from Lonza and cultured in Keratinocyte Growth Medium (KGM-Gold) and Fibroblast Growth Medium (FGM-2) (Lonza Group, Basel, Switzerland), respectively, according to the manufacturer’s protocol, apart from using PBS solution instead of HEPES-buffered saline solution (HEPES-BSS). With fibroblasts, FGM-2 medium was used to neutralize the trypsin instead of trypsin neutralizing solution (TNS). Fibroblasts were used for the models at passage 5–6 (P5-6), and the keratinocytes at P5. iPSC-derived neurons were prepared and cultured as described previously [21, 27]. In short, the human iPSC line LUMC0031iCTRL08 (Leids Universitair Medisch Centrum iPSC core facility) was cultured on Geltrex-coated dishes and further differentiated as nociceptors and neuronal spheres using a protocol based on the work of Chambers et al. [21, 27].

### Skin model preparation

Skin models (three batches of samples: *N* = 18, *N* = 10, *N* = 10) were made by first adding 200 μl of fibrin hydrogel produced as previously described [21], containing fibroblasts with a density of 500,000 cells/ml into the bottom of the cell culture inserts (BRND782807, VWR, Radnor, PA, USA). Aprotinin (Sigma-Aldrich) 100 μg/ml supplement was used in the fibrin and in all media used with fibrin gel to prevent hydrogel degradation. The fibroblast-laden fibrin gel was then incubated for 15 min at 37 °C for jellification and culture-submerged in FGM-2 medium, at 37 °C and 5% CO_2_.

The next day, keratinocytes were seeded at a density of 200,000 cells/cm^2^ on top of the fibrin gels. The constructs were then cultured submerged in the keratinization medium for 3 days before lifting it into the air–liquid-interface (ALI). The medium was changed every 2–3 days for 28 days. The samples were fixated with 4% paraformaldehyde (PFA) in PBS at day 21 calculated from the keratinocyte seeding. The compositions of the media are shown in Table S1.

### Innervated skin model preparation

**Fig. 2:**
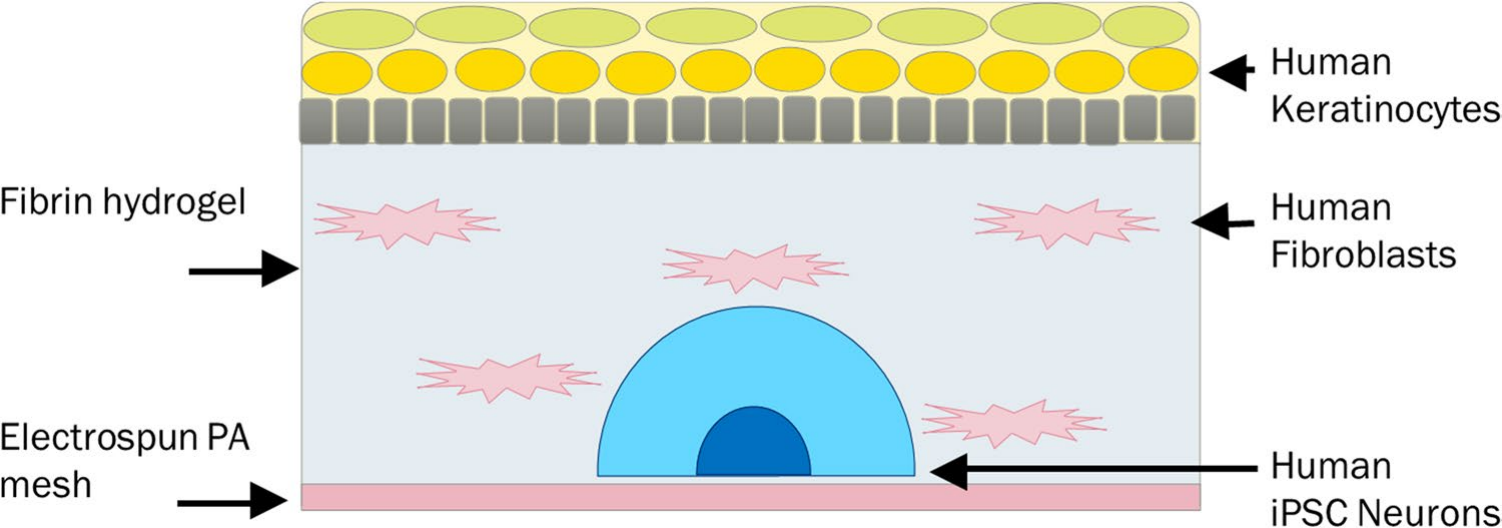
Schematic illustration of the innervated skin model

Innervated skin models (three batches of samples: *N* = 16, *N* = 10, *N* = 20) were prepared by seeding the iPSC-derived neurons as single spheroids on the electrospun scaffolds coated with mouse-laminin 1, as described previously [21]. These neuronal spheres were then cultured in neuronal medium with nerve growth factor (NGF) supplement of 1 μl/ml for 5 days before adding the fibrin gel with the fibroblasts as described above. Neuronal model medium was used for the culture from this step forward. The compositions of the neuronal and neuronal model media are shown in Table S1. The iPSC-derived neurons and the fibroblasts were culture-submerged for 1 day before seeding the keratinocytes on the top of the gel. The construct was then culture-submerged for 3 days before lifting it into ALI. The samples were fixated with 4% PFA at days 14, 21, or 28 calculated from the keratinocyte seeding. A schematic illustration of the innervated skin model is shown in Fig. 2.

### Capsaicin test

Capsaicin test was performed by adding capsaicin with 262 mM concentration on top of the innervated skin model at day 27 from the keratinocyte seeding, and incubated for 30 min (*N* = 5). After the exposure, the capsaicin solution was aspirated, the 3D in vitro model was washed with PBS three times, and it was returned to the ALI-culture for 24 h before fixation. The capsaicin powder was first dissolved into 100% ethanol and further diluted into the neuronal model medium. The final EtOH concentration in the medium solution was 0.26%. In the control sample, the steps were conducted the same way, but with only the neuronal model medium with 0.26% of EtOH added without the capsaicin.

### Tattoo testing

The tattoo testing was performed at day 28 by rapidly puncturing the epidermis with a 32-G needle connected to a 1-ml syringe containing a black tattoo ink bought from a tattoo parlor (*N* = 3). The samples were imaged with a stereomicroscope (SMZ25, Nikon, Tokyo, Japan) at days 0, 1, 5, and 6. The models were culture-submerged in the medium between days 5 and 6 and fixated after imaging at day 6.

### Sample processing

For immunohistochemistry (IHC), the PFA-fixed samples were embedded in optimal cutting temperature (OCT) medium and frozen in liquid nitrogen, then cryosectioned using the Kawamoto cryosectioning tape (C-MK001-B3, Section-Lab, Kawamoto, Osaka, Japan). Samples used for imaging as a whole in the capsaicin test were left unfrozen and stored in PBS in 4 °C after fixation until IHC staining. Immunohistochemical staining was performed with the antibodies presented in Table S2. After fixation, samples were washed three times with PBS, and permeabilized by incubating them 15–30 min with 0.1% Triton X-100. Then, the samples were washed 3 times with PBS and blocked for 1 h for the sections, and 24 h for the whole constructs using PBS-based blocking buffer containing 0.05% Tween 20, 5% goat serum, and 1% bovine serum albumin (BSA). After this, the primary antibody diluted in the blocking buffer was added and the samples were incubated 1 h or 24 h before washing 3 times with wash buffer (0.05% Tween 20 and 1% BSA in PBS), after which the secondary antibody diluted in wash buffer was added and incubated for 1 h or 24 h. After this, phalloidin and DAPI stainings were made. Negative control samples were processed otherwise similarly, but they were stained with the secondary antibody alone.

For formalin-fixed paraffin-embedded (FFPE) samples, the paraffin embedding was performed after dehydrating the samples in 50–70–96–100–100% EtOH for 30 min each, after which in xylene for 30 min for 2 times, and finally in paraffin for 1 h. Histological assessment was based on hematoxylin and eosin staining, and Masson’s trichrome staining (both from Thermo Fisher Scientific, Waltham, MA, USA), which were performed using the manufacturer’s instructions. The controls used for the histological and IHC samples were FFPE samples of human skin from young, healthy-skinned adult obtained from the pathology department of Maastricht University Medical Center (MUMC +).

The sections were imaged with a Slide Scanner microscope (TI-E, Nikon, Tokyo, Japan), manual microscope (TI-S, Nikon), or with a bright-field microscope (Leica, Wetzlar, Germany). The whole samples used in the capsaicin test were imaged using the confocal microscope (TCS SP8 STED, Leica, Wetzlar, Germany). The images were processed using Fiji/ImageJ software.

### Lipid analysis by mass spectrometry imaging

For assessing the suitability of the model for mass spectrometry imaging, we performed lipid analysis. To be applicable in the use of cosmetic testing, for example, the absorption and distribution of the compound studies could be performed using mass spectrometry imaging (MSI). The samples were left in ALI-culture for 24 h. The samples (*N* = 6) were embedded in 10% gelatin, flash frozen in liquid nitrogen, and cryosectioned in 10-μm sections using double-sided copper tape for MSI.

DHB matrix 15 mg/ml in 70% MeOH was sprayed with a HTX TM-Sprayer (HTX Imaging, Chapel Hill) for sample preparation (60 °C, 8 passes, 0.12 ml/min, flow rate, 1200 mm/min velocity, 0 s dry time, and 40 mm nozzle height). A SYNAPT G2Si instrument was used to acquire the imaging data over the mass range *m*/*z* 200–1400 in positive ion mode using the sensitivity mode and a scan time of 1 s per pixel. The Nd:YAG MALDI laser was operated at a firing rate of 1000 Hz with a lateral resolution of 50 μm. Acquired data were processed and analyzed using HDI Imaging software V1.4 (Waters Corporation) by extracting the 1000 most abundant ions with the following settings: *m*/*z* window = 0.02 Da and MS resolution = 15,000. Intensities were normalized by total ion count (TIC).

## Results

### Histological and immunohistochemical characterization

**Fig. 3:**
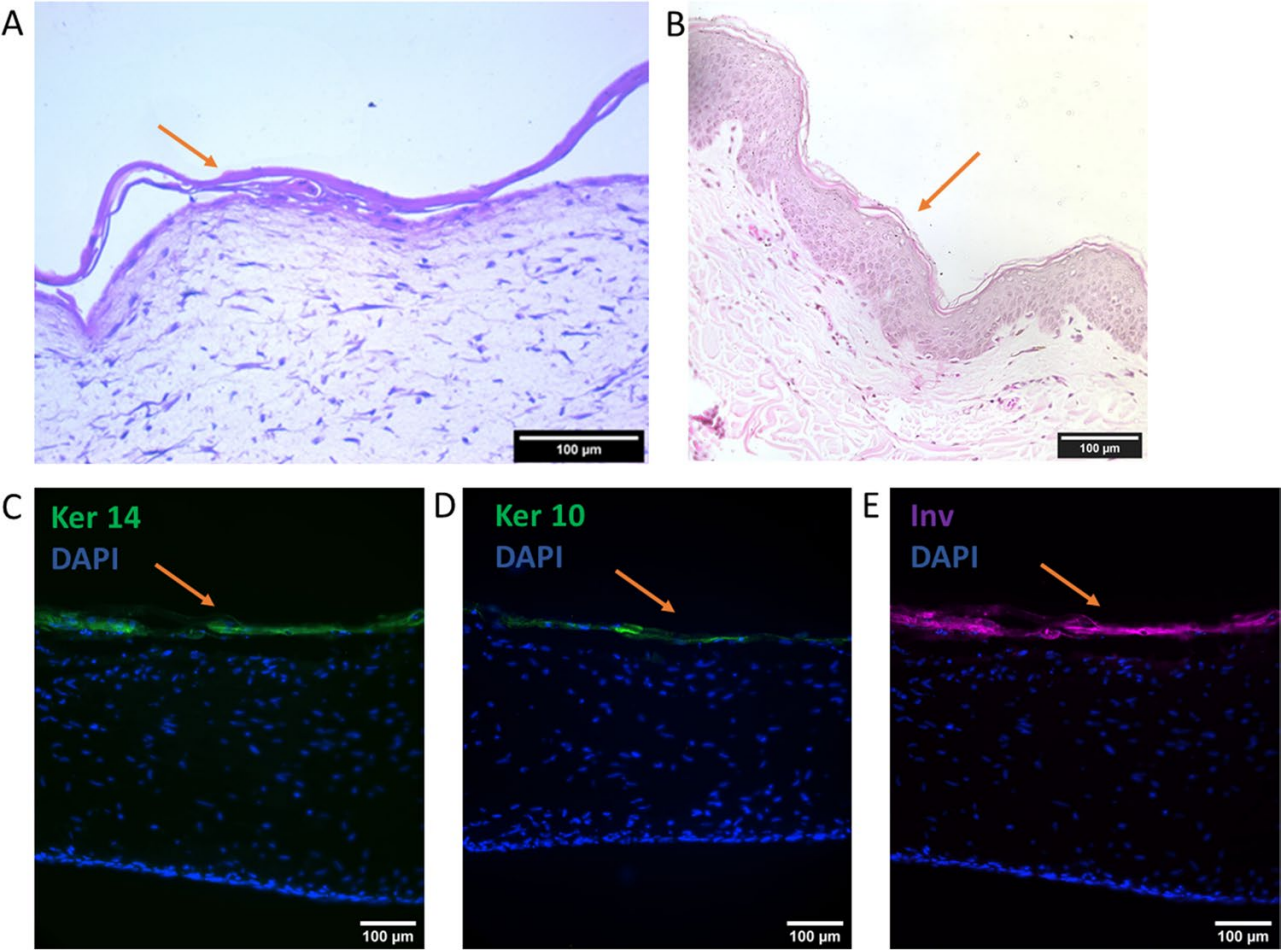
Histological and immunohistochemical characterization of the skin model at day 21 shows dermal and epidermal components in H&E (**A**). H&E-stained section of human FFPE skin sample (**B**). Keratinization markers keratins 14 and 10, and involucrin are present in the skin model (**C**, **D**). Ker 10 = keratin 10, Ker 14 = keratin 14, Inv = involucrin. Arrow is pointing at the epidermis. Scale bars: 100 µm

Histological characterization of the skin model at day 21 showed epidermis formation and stratification. The development of epidermal layers was visible, and the morphology became flaky towards the top layers of the epidermis, resembling the stratum corneum. However, the stratum spinosum remained thin in the skin model, and the morphology of the stratum basale was less cuboidal compared to the stratum basale of the native human skin. This could be due to improper formation of the basement membrane between the epidermal and dermal layers, which could be a result of insufficient collagen production from the fibroblasts or lacking the required signaling from adjacent tissues that would be present in vivo. Between the basal and top layers, a layer resembling stratum granulosum was observed, similarly to human skin. Fibroblast density seemed larger in the skin model compared to human skin, indicating the vitality of these cells during culture. In immunohistochemical staining, the skin model expressed the keratinization markers keratin 14 (in the basal layer), keratin 10, and involucrin (in the upper layers) in the epidermis (Fig. 3). The correct localization of keratinization markers keratin 14 and involucrin was seen already at day 14 after keratinocyte seeding, suggesting this culture time would be enough for the skin model to form keratinized epidermis (Figures S1 and S2).

**Fig. 4:**
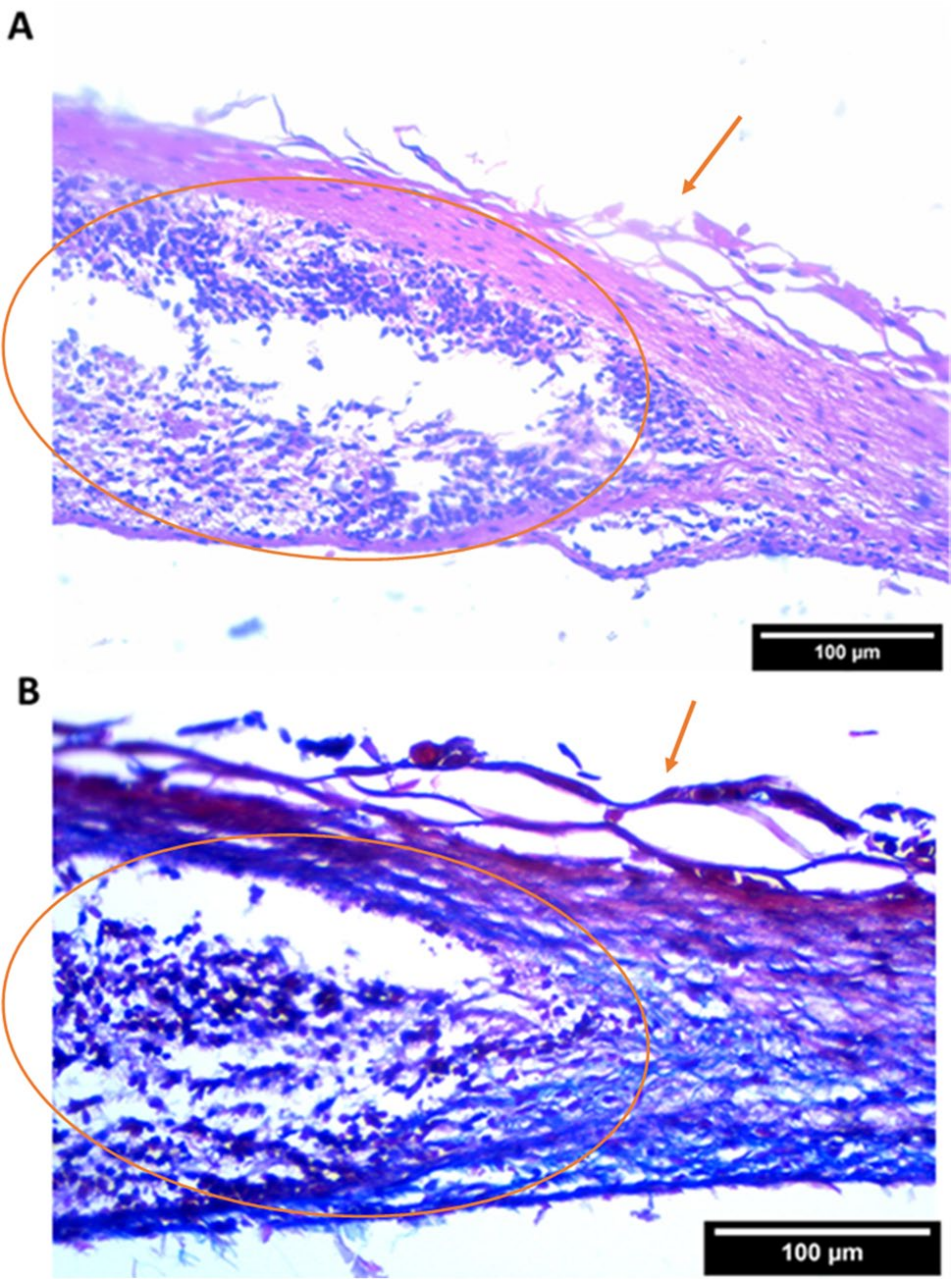
**A** H&E staining at day 21 from keratinocyte seeding shows the neuronal sphere and surrounding skin component. **B** Masson’s trichrome staining at day 21 shows blue collagenated dermal component. Arrows are pointing at the epidermis and the circles are around the neuronal sphere. Scale bars: 100 µm

**Fig. 5:**
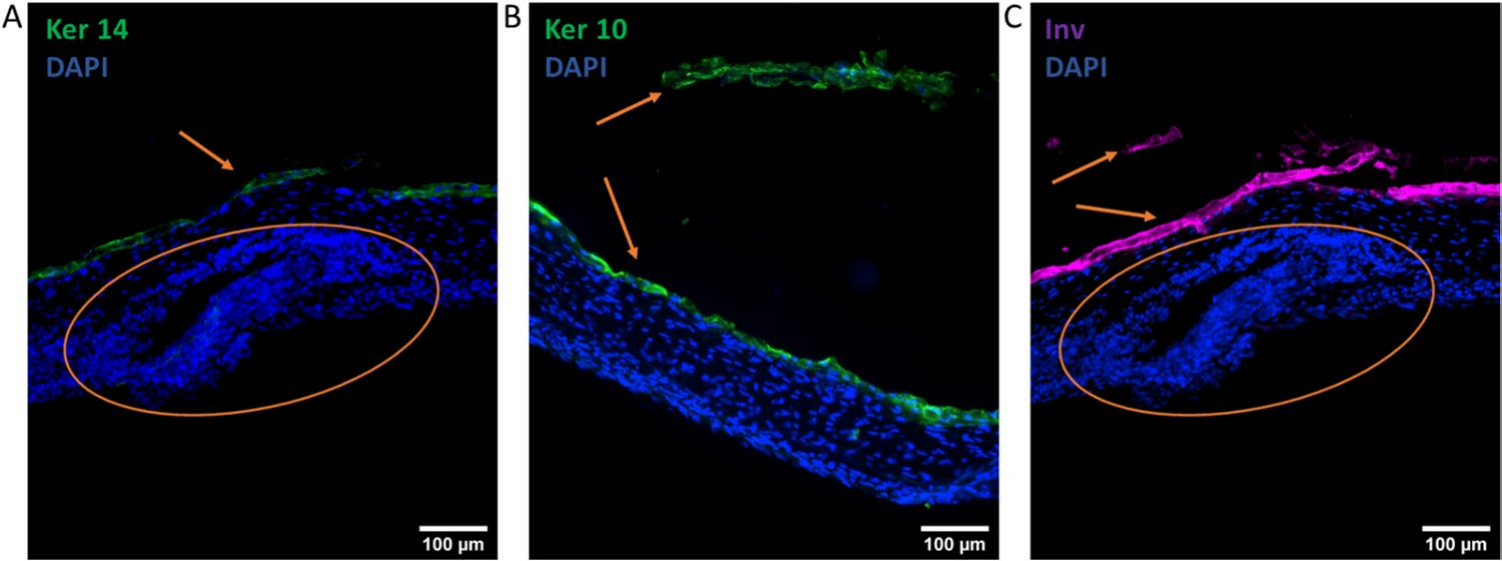
Immunofluorescent staining of the innervated skin model (**A–C**) at day 28 shows the expression of the keratinization markers keratins 14 (Ker 14) and 10 (Ker 10), and involucrin (inv). Arrows are pointing at the (partially detached) epidermis, and circles are drawn around the neuronal sphere. Scale bars: 100 µm

**Fig. 6:**
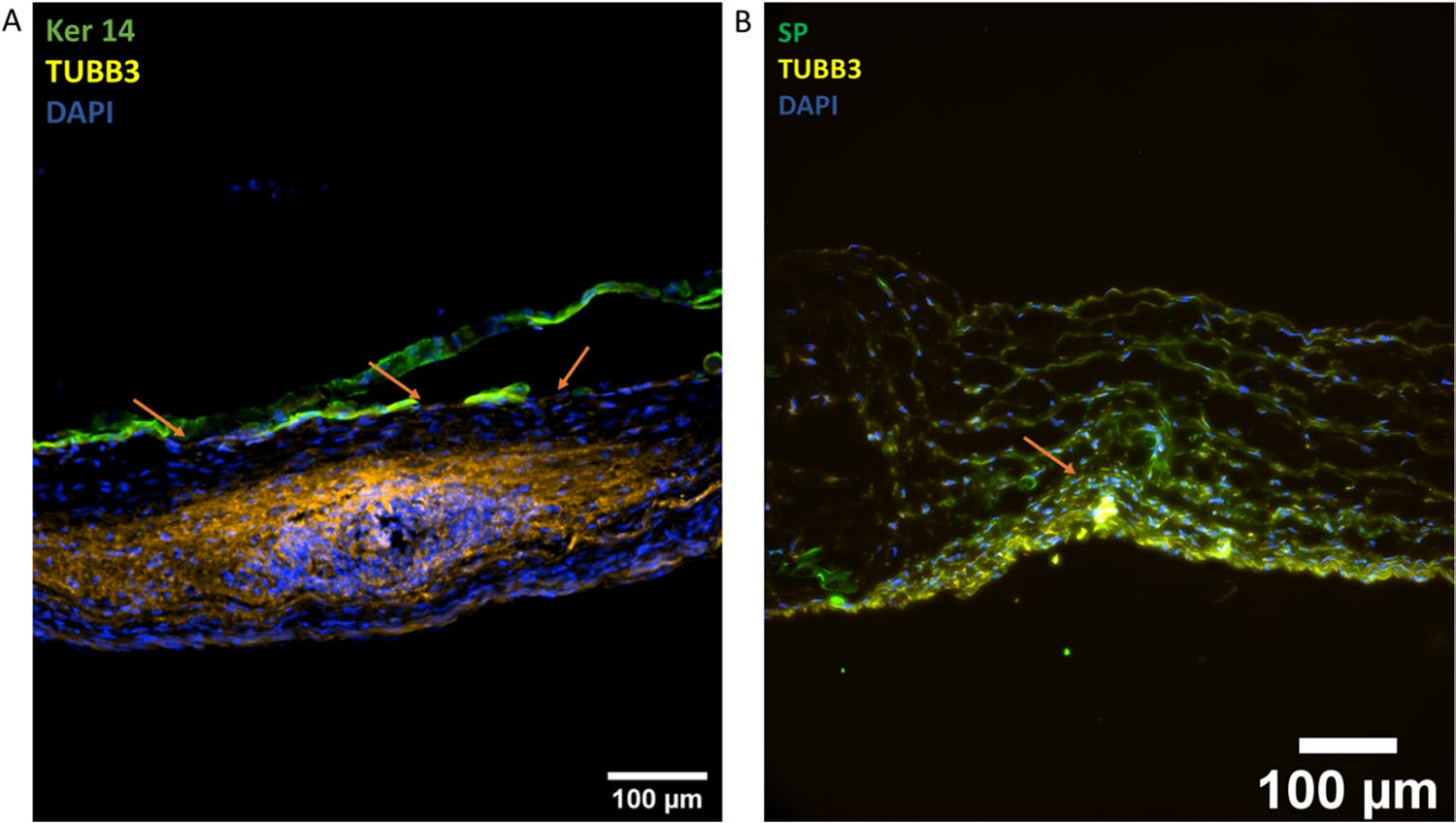
**A** The iPSC sensory neurons reached the epidermis (arrows) in the innervated skin model at day 28. **B** Neuronal markers substance P (SP) and tubulin 3 (TUBB3) are expressed mostly in the neuronal sphere (arrow), but they are seen also in the axons spreading throughout the scaffold. Ker 14 = keratin 14. Scale bars: 100 µm

In the innervated skin model, histological staining with H&E and Masson’s trichrome at day 21 showed axonal outgrowth into the scaffolds, flaky epidermis, and a collagen-containing dermal component. The blue-stained collagen fibers could be observed also in the upper parts of the scaffolds between the axons in the Masson’s trichrome–stained histological sections, indicating the viability of the fibroblasts within the dermal component (Fig. 4). Further characterization at day 28 of the innervated skin model by immunofluorescent staining showed the successful keratinization process of the model (Figs. 5 and 6), similar to the skin model (Fig. 3). The alignment of the scaffold’s nanofibers was also confirmed (Fig. 1).

When staining the innervated skin models with the neuronal marker tubulin β3 and the epidermal marker keratin 14, axonal spreading throughout the scaffolds and into the epidermis was observed. When staining the models together with the neuroinflammatory marker substance P and neuronal marker tubulin β3, co-localization of these markers in the axonal area was observed. The expression of substance P was higher in the neuronal sphere than in the axons outside the sphere, where the neurons were more dispersed (Fig. 6).

### Capsaicin test

To evaluate the suitability of our model for in vitro testing, we chose to stimulate it with capsaicin. By exposing the model to capsaicin at 262 mM concentration for 30 min, we aimed for inducing axonal loss in the epidermal area of the model, and substance P depletion in that area as consequence of the axonal loss. We assumed that imaging the construct 24 h after the treatment would reveal possible effects of the capsaicin in a more reliable manner, since the neurons would have time to degrade. However, it is to be noted that the actual concentration of the capsaicin in the plaster used in the clinics is higher, since its solubility to EtOH is lower than in a cream formulation.

**Fig. 7:**
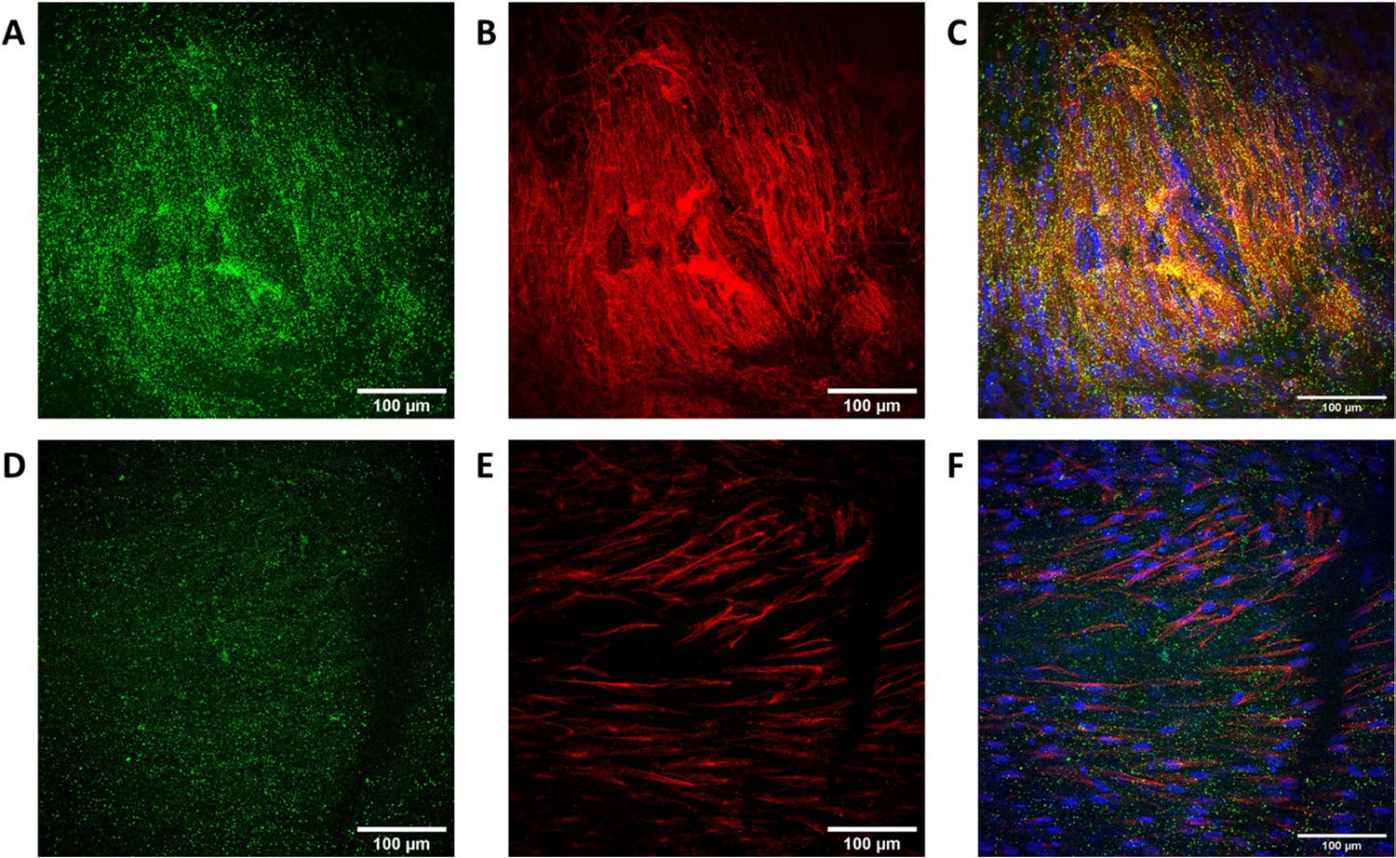
Innervated skin model shows partial loss of substance P (in green) and tubulin β3 (in red) after capsaicin treatment (**D–F**) compared to the control sample (**A–C**). Images **C** and **F** show merged image of substance P and tubulin β3. Scale bars: 100 µm

In capsaicin-treated samples, the axonal loss and substance P depletion could be seen to some extent, despite the variability within the samples. In some of the capsaicin-treated samples, it was challenging to find a spot with vital neurons to image. The same effect was not observed in non-treated samples (Figs. 7 and S3). For comparison, images of the same capsaicin tests performed on neurons seeded on laminin 1–coated coverslips showed depletion of a neuropeptide calcitonin gene–related peptide (CGRP) and neuronal marker tubulin β3 when imaged at 24 h after 30-min exposure to capsaicin when compared to its control sample (Figure S4). The samples did not show signs of shrinkage macroscopically or microscopically, and the electrospun mesh stabilized the fibrin gel. However, in the last end point at day 28, the construct was slightly lower in height than in previous timepoints.

### Tattoo ink test

**Fig. 8:**
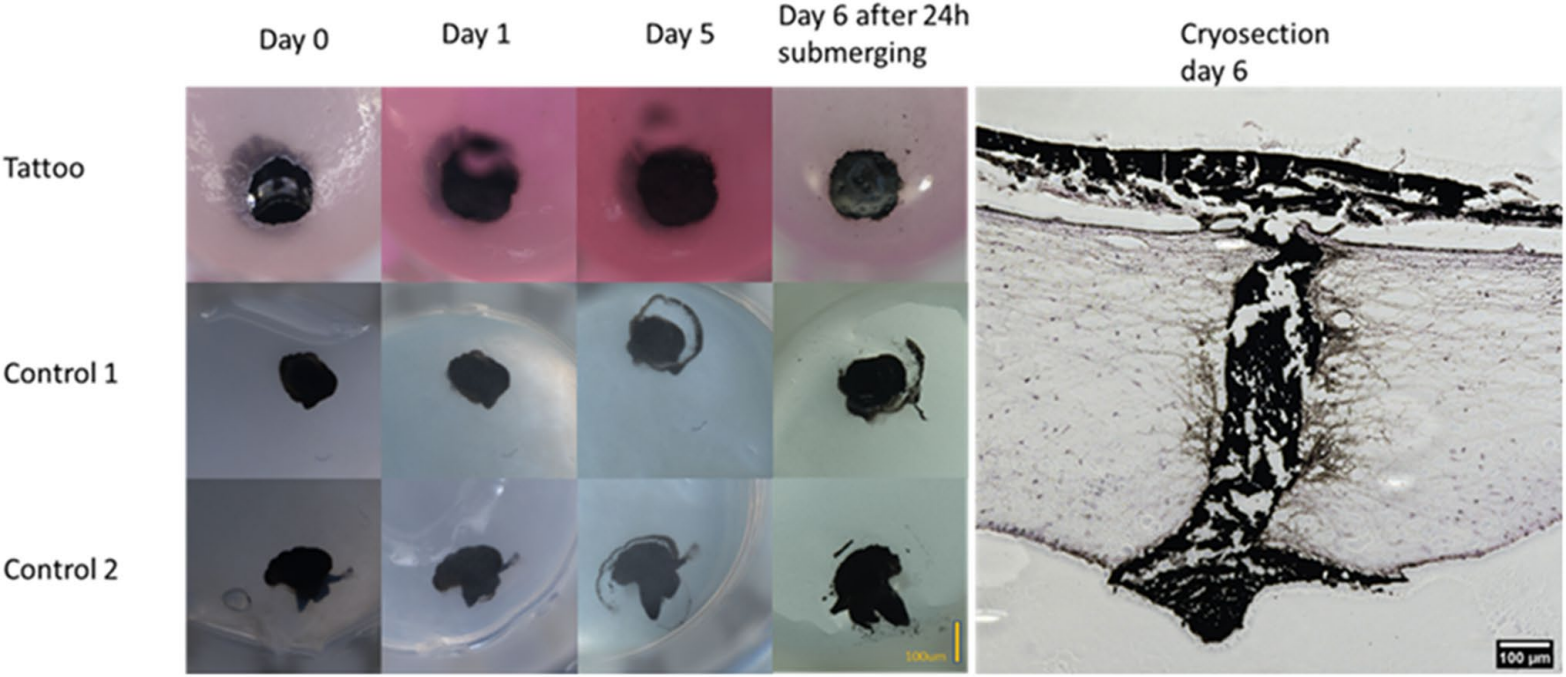
The stereomicroscope images of the tattoo test and the cryosection of the tattooed skin model demonstrate the retention of the tattoo ink in the model and in the controls for 6 days. The control samples are produced identically to the tattoo test into fibrin gel and incubated in PBS using otherwise similar protocol. Scale bars: 100 μm

The aim of the tattoo test was to present the ability of the skin model to retain a tattoo ink injected intradermally. With this ability, the skin model could be used to test intracutaneous compounds in the future. The tattoo ink stayed in place in the model, as shown in Fig. 8. We did not observe the tattoo ink to have leaked into the culture medium/PBS. In human skin, the tattoo ink also stayed in place; with this ability, it could be possible to also compare, for example, the viability of the cells adjacent to the tattoo ink to the ones located more distally.

### Lipid visualization with mass spectrometry imaging

To test the ability of the skin model to retain skin cell properties and function as a tool for future cosmetic testing, we performed MSI experiments for lipid visualization, which showed the presence of cell-membrane lipid species into the dermal component (Figure S5). When able to visualize the construct in MSI, it would be possible to add different cream formulations on the construct, for example, and measure their absorption in the skin model.

## Discussion

In this study, we have described an innervated skin platform that could be used in vitro for dermatological research. Unlike otherwise similar innervated skin models described in the literature, our model is made with human cells [1, 12]. This avoids the need of the use of animal-derived cells, such as rat dorsal root ganglion cells (DRGs), and of human skin obtained from a donor [10]. Muller et al. reported an interesting innervated human skin model. However, they were unable to get the iPSC-derived neurons to reach the epidermis without adding rat- or human iPSC-derived Schwann cells into the culture [1]. In our model, the iPSC neurons spread throughout the scaffold without the addition of Schwann cells, and the epidermis of our model showed a panel of keratinization markers. Blais et al. described a sophisticated innervated wound healing model, yet animal-derived sensory neurons were used [11].

In this study, we demonstrated that it is possible to develop a human innervated skin model from commercially available primary keratinocytes and fibroblasts, together with iPSC-derived sensory neurons. The fibrin gel and electrospun PEOT/PBT mesh were suitable scaffolds for this model. It was also possible to leave the neuronal component out, resulting into a skin model suitable for cosmetic testing or as an alternative for a 2D co-culture of keratinocytes and fibroblasts. Shrinkage of the model, which has been a problem in collagen-based scaffolds, was not an issue when using an electrospun mesh as a stabilizer of the fibrin gel along with its main function as nerve growth conduit. The skin or skin neuron models did not show shrinkage macroscopically or microscopically when using a fibrin gel.

In future studies, the model here described could be used to also study skin cancer. For example, by adding malignant melanocytes or malignant keratinocytes into the model, it could be possible to mimic melanoma or keratinocyte carcinoma in vitro. By replacing the healthy skin cells with diseased ones, the model could be suitable for studying dermatological conditions. Using patient-derived (malignant) skin cells instead of commercial ones, the model could be used for testing the patient’s response for different treatment options, thus opening the route towards personalized dermatological research. It would be interesting to incorporate immune cells into the model, thus assessing also neuroinflammation in skin sensitization [15]. Seeding the neurons as spheroids allows the axons to spread in the model, and therefore could be used to study axonal regeneration in a wound healing model.

Even though the epidermis of our models did express keratinization markers, it is still not fully comparable to the human skin. The stratum basale had a less defined cuboid cell shape than in the human skin, and no proper multi-layered stratum spinosum could be seen. This could possibly be due to improper basement membrane formation. The basement membrane is a thin complex formed by the stratum basale of the epidermal keratinocytes and the dermal fibroblasts. Its core consists of a thin collagen IV lattice, into which proteins, secreted by the basal keratinocytes and upper dermis fibroblasts, such as integrins and laminin 332 (former laminin 5) are attached. Into these functional sites, the keratinocytes of the stratum basale anchor forming hemidesmosome junctions. These junctions are important for the epidermis formation, since they guide the cellular cytoskeleton organization through keratin intermediate filaments, leading to a signaling cascade eventually leading to the proper stratification of the epidermis. If the basal keratinocytes and fibroblasts are not able to form a sufficiently strong basement membrane, the stratification cannot occur [28]. Another possible explanation for the suboptimal stratification could be the low amount of keratinocyte stem cells in the keratinocytes used in the models [25]. Despite the apparent poor stratification, testing with capsaicin still provides confirmation on the possibilities of topical drug testing using this model.

The iPSC-derived sensory neurons reached the epidermis in our model and seemed to form linear structures in the dermis, resembling the neuronal architecture present in the native human skin [29, 30]. Addition of Schwann cells to the neuronal component could bring the model closer to the native innervated skin. Even though we cultured the models for 21 or 28 days, we observed that the culture time of 14 days was enough (Figs. S1 and S2). Culturing the model for 21 or 28 days did not seem to further mature the skin or the neuronal component, and the thickness of the model seemed to decrease along with the culture time.

If patient-specific iPSC-derived neurons would be used, the platform described in this study could be used for both neurological or dermatological research and drug discovery. However, the advantage of using commercially available pooled primary keratinocytes and fibroblasts is to guarantee reproducibility and to reduce variation between batches. This also makes the production of the 3D in vitro models independent of the patient cell source, thus being available for more laboratories, not only for those connected with a hospital.

In the model described in this study, we preferred using non-animal-derived cells. However, the model is not completely deprived of animal-based products, since FBS and Geltrex were used. Also, the primary cells used in this model are of neonatal origin, which may raise ethical questions on whether male circumcision is an acceptable way of obtaining cells for research use. Another drawback of these cells is their male origin, since the iPSC neurons used were female [21]. In the future, our aim is to develop an innervated skin model by using solely iPSCs of the same sex and synthetic hydrogels to substitute animal-derived products.

To further develop our model, it would be important to enhance the epidermis stratification. This could be done, for example, by coating the fibrin gel with basement membrane proteins, such as collagen IV or laminin 322 [31]. This could lead to better attachment and polarization of the keratinocytes in the stratum basale, and therefore promote the stratification. One interesting approach would also be to have electrospun scaffolds made of collagen and coated with laminin 322 between the keratinocytes and the fibrin gel. Bioprinting strategies could be used to improve stratification of these components to make it correspond better to the human skin. Further validating the model using quantitating tests, such as ELISA assays to quantify the functional presence of skin and neural markers, barrier testing, and electroconductivity could be performed, as well as testing the model’s ability for wound healing model.

## Conclusion

In conclusion, the human innervated skin model developed in this study is highly feasible and versatile for in vitro evaluation, therefore limiting the need for in vivo animal testing.

## Supplementary Information

Below is the link to the electronic supplementary material.

Supplementary file1 (DOCX 5.45 MB)
