# Supplementary material for: An innervated skin 3D in vitro model for dermatological research
Source: In Vitro Model. 2022 Jun 10;2(3-4):113–21. doi: 10.1007/s44164-022-00021-0 (PMC11756442; doi:10.1007/s44164-022-00021-0)
Supplement: Supplementary file 1 — Supplementary file1 (DOCX 5.45 MB) [file 44164_2022_21_MOESM1_ESM.docx]

**Supporting Information**

**An innervated skin 3D in vitro model for dermatological research**

Emma Rousi^1,2^, Afonso Malheiro^1^, Abhishek Harichandan^1^, Ronny Mohren³, Ana Filipa Lourenço^1^, Carlos Mota^1^, Berta Cillero-Pastor^3^, Paul Wieringa^1^, Lorenzo Moroni^1^

*¹MERLN Institute for Technology Inspired Regenerative Medicine, Department of Complex Tissue Regeneration, Maastricht, The Netherlands*

*²University of Turku, Institute of Clinical Medicine, Department of Surgery, Turku, Finland*

*³M4i division of Imaging Mass Spectrometry, Maastricht University, Maastricht, The Netherlands*


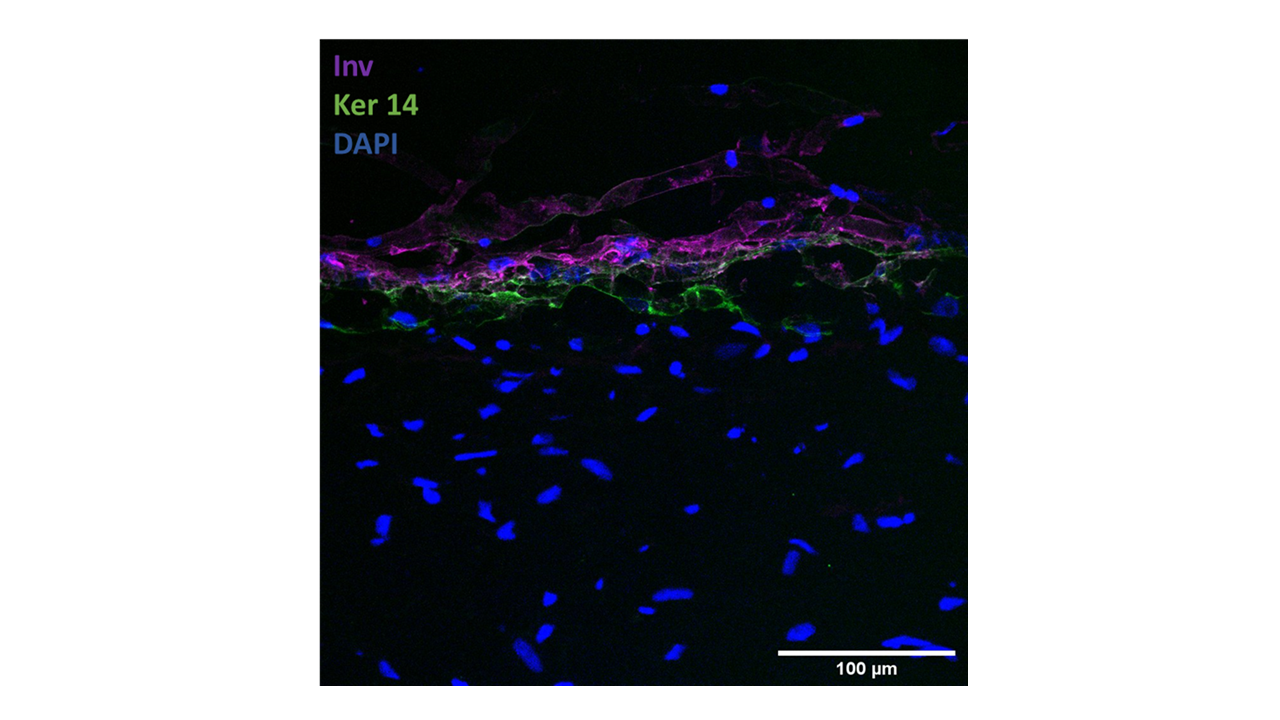


**Figure S1**. The localization of keratinization markers keratin 14 (Ker 14) and involucrin (Inv) appears correct in the 3D in vitro skin model already at day 14 after keratinocyte seeding. Scale bar: 100 µm.


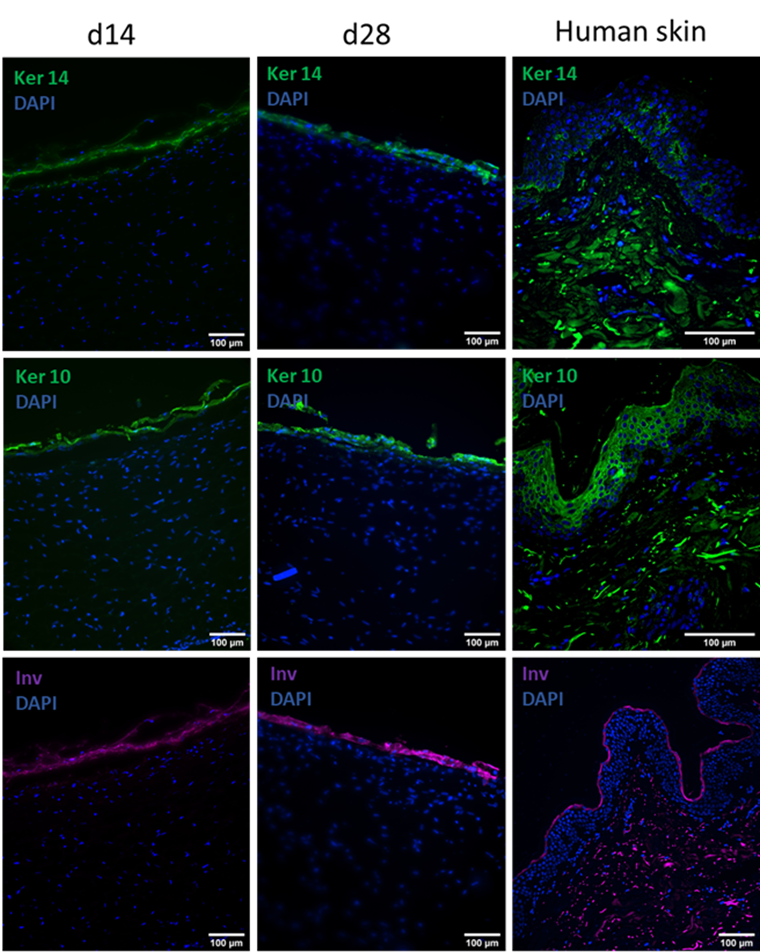


**Figure S2.** Keratinization process at days 14 and 28 on the fabricated 3D in vitro models compared with human skin controls. All keratinization markers are present already at day 14. In human skin samples, staining of keratins 14 and 10, and involucrin are seen also in the dermis. Scale bars: 100 µm.


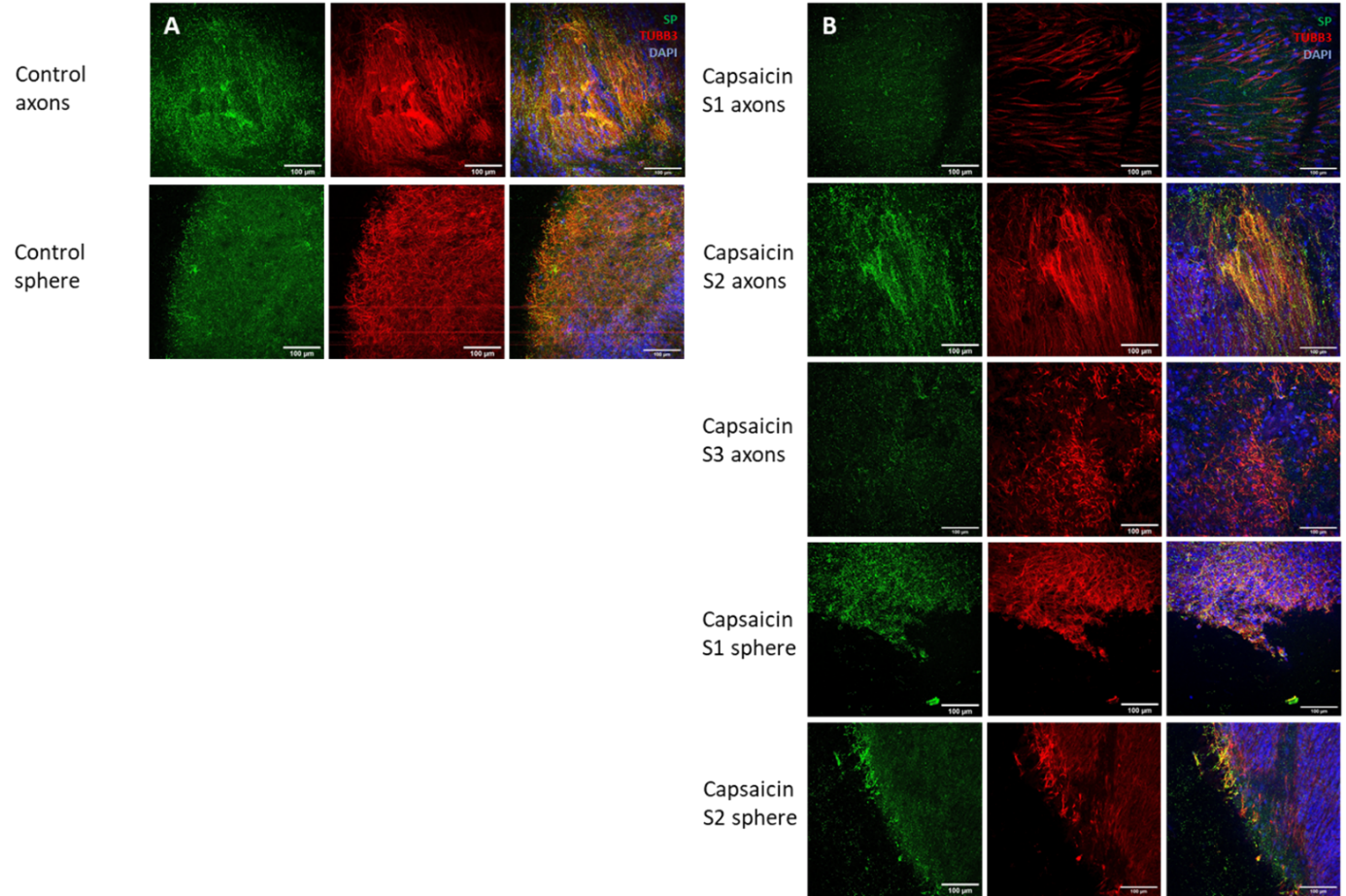


**Figure S3.** The control sample of the innervated skin model shows vital axons spreading and co-located Substance P (SP) expression. The capsaicin treated samples show sparse, damaged axons and weaker SP expression. Scale bars: 100 µm.


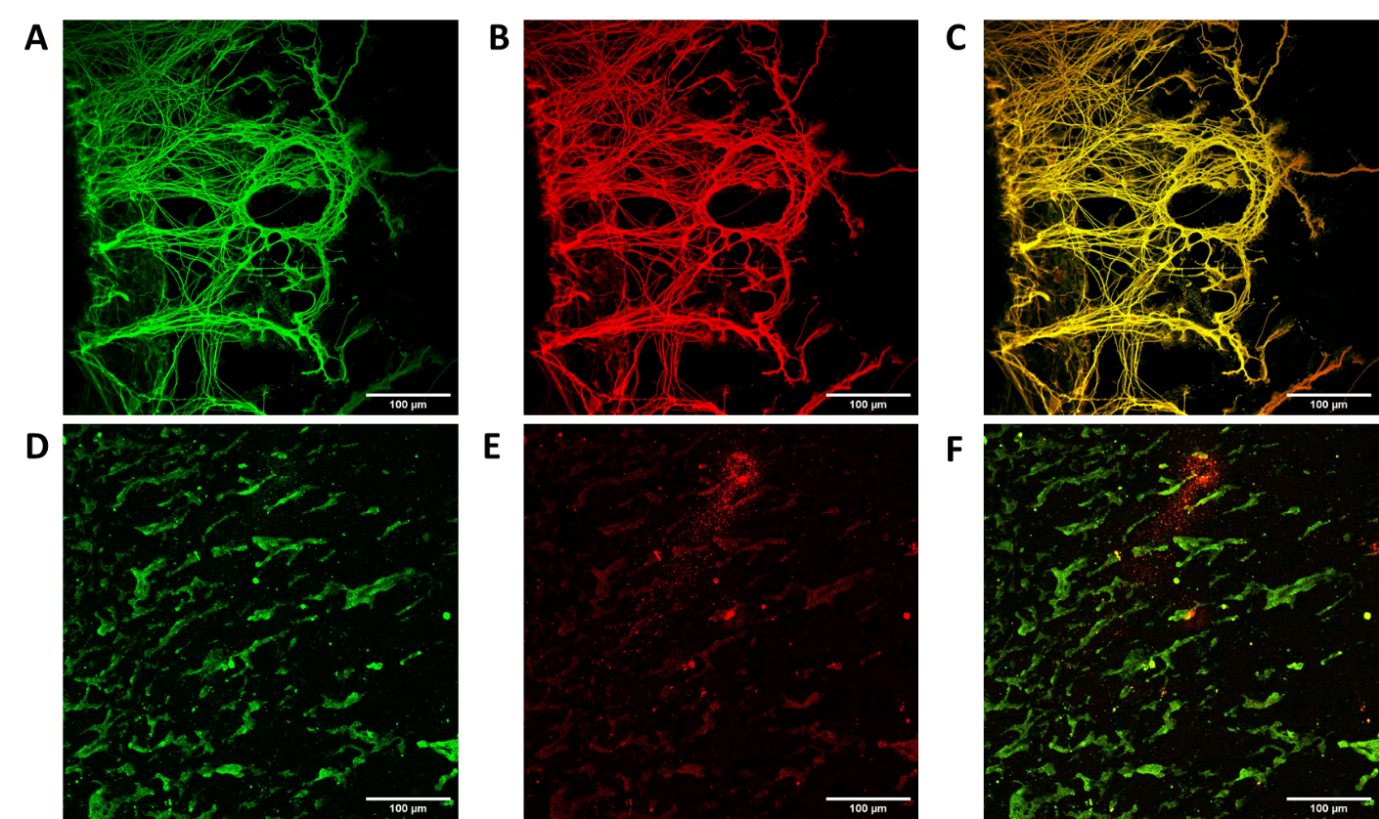


**Figure S4**. Neurons on coverslips treated with Capsaicin at 262 mM for 30 min (D-E) and imaged after 24h show depletion of Calcitonin gene-related peptide (CGRP, in green, A,D) and Tubulin β3 (Red, B,E) when compared to the control samples (A-C). Images C and F show merged images of CGRP and Tubulin β3. Scale bars: 100 µm.


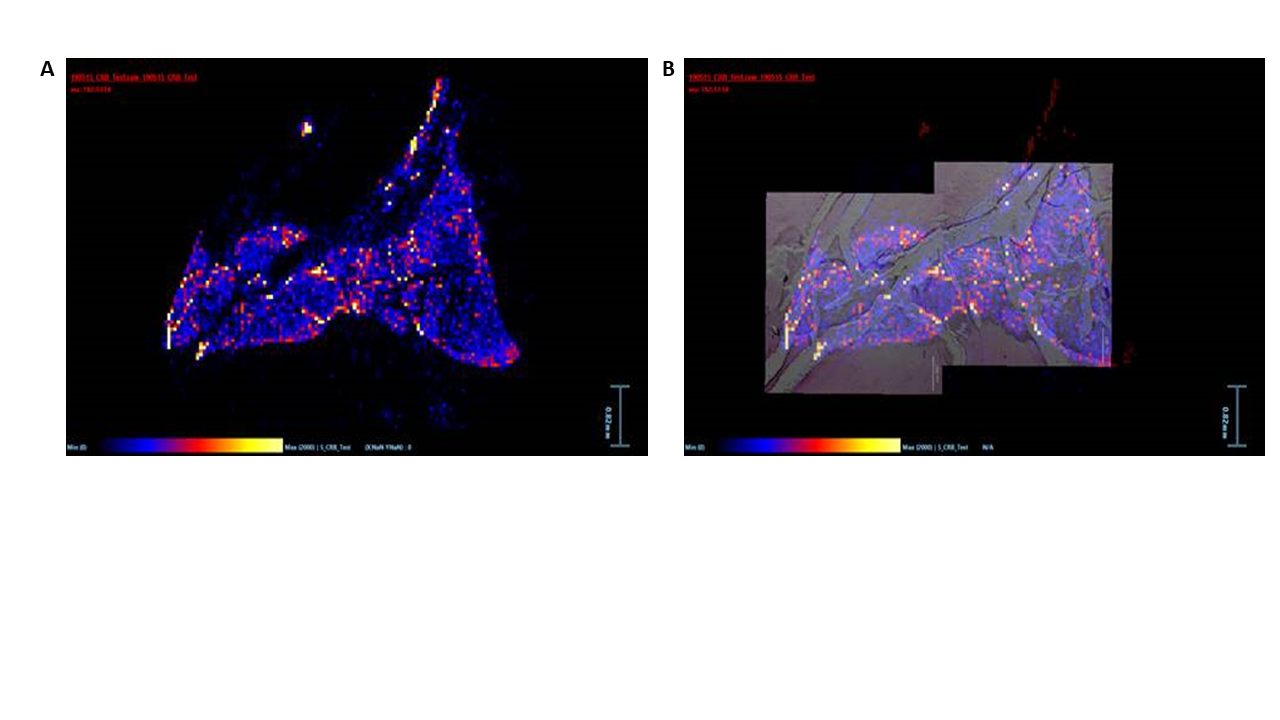


**Figure S5.** Mass spectrometry images of the skin model (**A**) alone and combined with H&E stained cryosection of the same sample (**B**) show the presence of lipid species into the dermal component (*m/z* 782.5, phosphatidylcholine as tentative assignment).

**Tables**

| **Keratinization Medium** | - DMEM (high glucose, GlutaMax) - 10% FBS - HKGS - 50ug/ml of Ascorbic Acid - PEN/STREP |
| --- | --- |
| **Neuronal Medium** | - Neurobasal medium - Glutamax (0.5mM) - N-21 supplement - Ascorbic acid (50ug/ml) - PEN/STREP - NGF (50 ng/ml) |
| **Neuronal Model Medium** | - Neurobasal medium - Glutamax (0.5mM) - N-12 supplement - Ascorbic acid (50ug/ml) - PEN/STREP - NGF (50 ng/ml) - HKGS |

**Table S1.** Culture media for skin and innervated skin 3D *in vitro* models. HKGS=human keratinocyte growth supplement, NGF=Nerve Growth Factor (both from Thermo Fisher Scientific).

| **Staining** | **1^st^ antibody/**  **species** | **Dilution** | **Vendor** | **2^nd^ antibody/ Alexa Fluor** | **Dilution** | **Vendor** |
| --- | --- | --- | --- | --- | --- | --- |
| **Substance P** | Mouse | 1:1000 | Abcam ab14184 | Goat anti mouse | 1:800 | Thermo Fisher Scientific |
| **Tubulin β3** | Mouse | 1:800 | Sigma-Aldrich T8578-200UL | Goat anti mouse | 1:800 | Thermo Fisher Scientific |
| **Tubulin β3** | Chicken | 1:800 | Bio-Techne  NB100-1612 | Goat anti chicken | 1:800 | Thermo Fisher Scientific |
| **Keratin 14** | Mouse | 1:200 | Abcam  ab7800 | Goat anti mouse | 1:800 | Thermo Fisher Scientific |
| **Keratin 10** | Mouse | 1:500 | Abcam  ab212546 | Goat anti mouse | 1:800 | Thermo Fisher Scientific |
| **Involucrin** | Rabbit | 1:100 | Abcam  ab181980 | Goat anti rabbit | 1:800 | Thermo Fisher Scientific |

**Table S2.** Antibodies used for immunohistochemical staining of the skin 3D *in vitro* models.
